# A Novel Chimeric Anti-PA Neutralizing Antibody for Postexposure Prophylaxis and Treatment of Anthrax

**DOI:** 10.1038/srep11776

**Published:** 2015-07-02

**Authors:** Siping Xiong, Qi Tang, Xudong Liang, Tingting Zhou, Jin Yang, Peng Liu, Ya Chen, Changjun Wang, Zhenqing Feng, Jin Zhu

**Affiliations:** 1 Department of Pathology, Nanjing Medical University, Nanjing 210029, China; 2 Huadong Medical Institute of Biotechniques, Nanjing 210002, China; 3 Key Laboratory of Antibody Technique of Ministry of Health, Nanjing Medical University, Nanjing 210029, China; 4 National Institute for Communicable Disease Control and Prevention, Chinese Center for Disease Control and Prevention, Beijing, 102206 China

## Abstract

Anthrax is a highly lethal infectious disease caused by the bacterium *Bacillus anthracis*, and the associated shock is closely related to the lethal toxin (LeTx) produced by the bacterium. The central role played by the 63 kDa protective antigen (PA63) region of LeTx in the pathophysiology of anthrax makes it an excellent therapeutic target. In the present study, a human/murine chimeric IgG mAb, hmPA6, was developed by inserting murine antibody variable regions into human constant regions using antibody engineering technology. hmPA6 expressed in 293F cells could neutralize LeTx both *in vitro* and *in vivo*. At a dose of 0.3 mg/kg, it could protect all tested rats from a lethal dose of LeTx. Even administration of 0.6 mg/kg hmPA6 48 h before LeTx challenge protected all tested rats. The results indicate that hmPA6 is a potential candidate for clinical application in anthrax treatment.

The bacterium *Bacillus anthracis*, which primarily affects livestock but can also infect humans, is the causative agent of anthrax, a zoonotic disease and bioterrorism threat^1^,^2^. *B. anthracis* spores can be used as bioterror agents in biological warfare. This threat has spurred significant efforts toward the development of countermeasures for anthrax, including anthrax vaccines and therapeutics^3^. However, vaccines are effective only for prevention^4^. Currently, therapeutic antibodies that target the anthrax toxin are under development and are designed to protect against the disease.

*B. anthracis* secretes a tripartite toxin comprising a protective antigen (PA), lethal factor (LF), and edema factor (EF)^5^. This is an A-B (or “binary”) bacterial toxin. PA is the “B” subunit, which is responsible for cell surface binding, while LF and EF are A subunits responsible for the enzymatic activity of the toxin^6^,^7^. PA combined with LF or EF constitutes the lethal toxin (LeTx) or edema toxin (EdTx), respectively^8^. The first step in cellular intoxication involves binding of an 83 kD form of PA (PA83) to specific cell surface receptors (ANTXR1^9^ and ANTXR2^10^). Following receptor binding, PA83 is cleaved after the Arg-Lys-Lys-Arg sequence at amino acid position 167 by a furin-family protease. This results in a 63 kDa form (PA63) that spontaneously oligomerizes to either a heptamer or an octamer^11^,^12^,^13^. Dissociation of the 20 kDa form (PA20) from PA83 allows PA63 to bind to either or both EF and LF. Then, oligomeric PA63-receptor complexes translocate LF or EF into the cytosol, where they promote intoxication^14^.

Previous studies have shown that PA63 inserts stably and irreversibly into lipid bilayers to form ion-permeable channels^15^,^16^. Other research has shown that the protease cleavage site deletion or mutation in PA83 prevents EF and LF binding^17^,^18^, and that for cells treated with lysosomotropic agents, the ability of PA to mediate the actions of EF or LF is blocked^19^. Nasal immunization of mice with a mixture of PA63, LF, and a poly-γ-d-glutamic acid conjugate have been shown to exhibit strong antibody responses against all three antigens^20^. Thus, PA63 seems to be an ideal target fragment for antibody generation and selection.

Since passive immunization with protective antibodies can provide immediate and extensive protection independent of the host response, it is an attractive option to enhance the current postexposure treatment of anthrax. Especially with regard to biodefense, it is considered the primary available therapeutic measure^21^. During the past 10 years, extensive research has focused on development of therapeutic antibodies to target the main virulence factors of anthrax, namely, PA, LF, EF, and capsule^22^,^23^,^24^,^25^,^26^,^27^,^28^,^29^,^30^,^31^. Among these, PA plays a central role in the pathophysiology of anthrax and is an excellent therapeutic target. Further, PA63 is the most important part of PA.

In the present study, we developed murine IgG neutralizing antibodies that directly target PA63. Then, we selected an ideal antibody from among these and genetically recombined it to form human/murine chimeric IgG (coded “hmPA6”). hmPA6 could specifically bind to PA63 and protect J774A.1 cells against LeTx challenge *in vitro*. Further, it protected Fischer 344 rats (F344) from LeTx after challenge.

## Results

### ELISA

**Figure 1:**
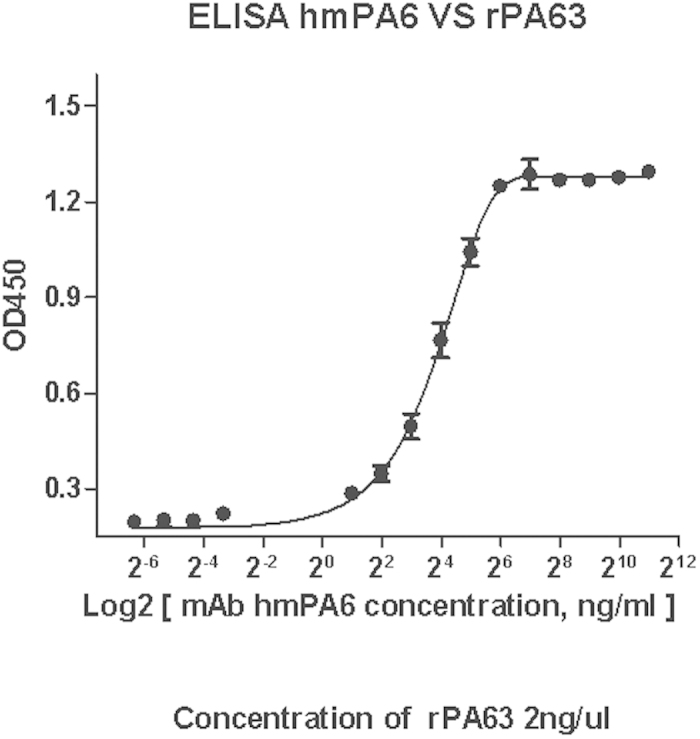
ELISA. rPA63 was used to coat ELISA plates. The wells were then incubated with serial dilutions of hmPA6, and the bound antibody was detected by the addition of peroxidase-conjugated goat anti-human antibody followed by tetramethylbenzidine substrate. OD450 = optical density at 450 nm.

ELISA was performed to test the binding sensitivity of hmPA6 to PA63. hmPA6 recognized rPA63 in a dose-dependent manner, and the graph of hmPA6 concentration and absorbance at 450 nm was a hyperbolic curve (Fig. 1).

### Western blot

**Figure 2:**
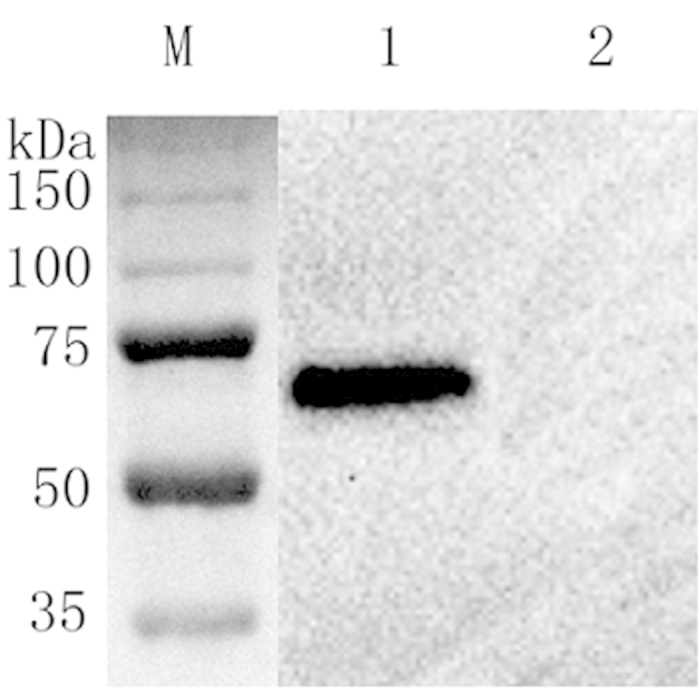
Western blot. M, molecular weight marker (NEB, USA); lane 1, lysates of rPA63 recombinant bacteria; lane 2, lysates of E. coli BL21.

Western blot analysis showed that hmPA6 could specifically recognize rPA63 (Fig. 2). No reaction was seen with the negative control.

### Immunoprecipitation

**Figure 3:**
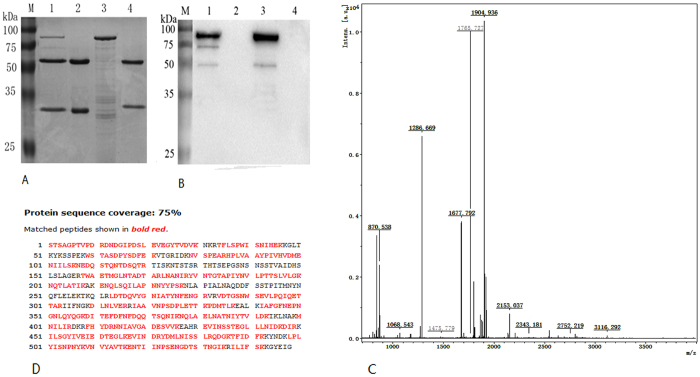
Immunoprecipitation (IP). **A**. 10% SDS-PAGE of IP. **B**. Western blot of IP. M, molecular weight marker (NEB, USA); lane 1, eluate of hmPA6 + a mixture of PA63 and PA83 from protein-A Sepharose; lane 2, hmPA6; lane 3, PA83; lane 4, eluate of anti-TLR4 chimeric IgG + a mixture of PA63 and PA83 from protein-A Sepharose. **C** and **D**. MS spectra of fragment ions from the 63 kDa protein. Five major (m/z = 870.539, 1267.709, 1479.797, 2034.010, and 2190.020) ions were detected. MS with protein database search: matched peptides are shown in red and boldface.

Immunoprecipitation was performed using PA83, which could be split to active PA63 using trypsin. A protein of about 63 kDa was detected on SDS-PAGE, and its sequence matched that of the *B. anthracis* protective antigen in the Swiss-Prot database (Fig. 3A,C,D). A 63 kDa membrane protein was also detected using a commercial anti-PA antibody (Fig. 3B), and this protein did not reaction with any other antibodies.

### Kinetics of binding

**Figure 4:**
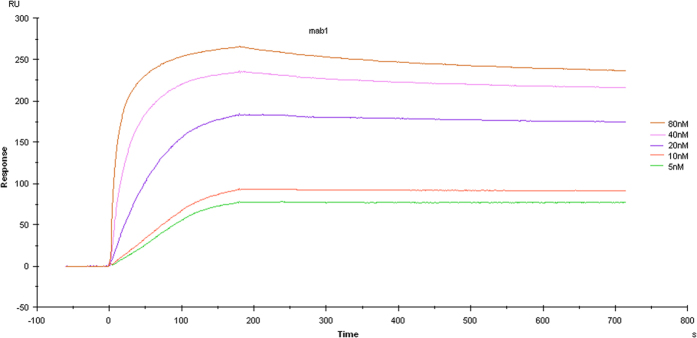
Affinity and kinetic assay. hmPA6 affinity and kinetics assays showed five curves with different concentrations of anti-PA IgG ranging from 5 to 80 nmol/L; Kd = 1.438 × 10^−10^ M with PA83 at 25 μg/mL.

The equilibrium dissociation constant (Kd) for hmPA6 was determined by BiaCoreX100 analysis. The rate constants kon and koff were evaluated directly from the BiaCoreX100 sensogram. The Kd was also determined using the BiaCoreX100. One striking feature of hmPA6 is its very slow off rate, which may explain its high affinity of 1.438 × 10^−10^ M (Fig. 4).

### *In vitro* LeTx neutralization assay

**Figure 5:**
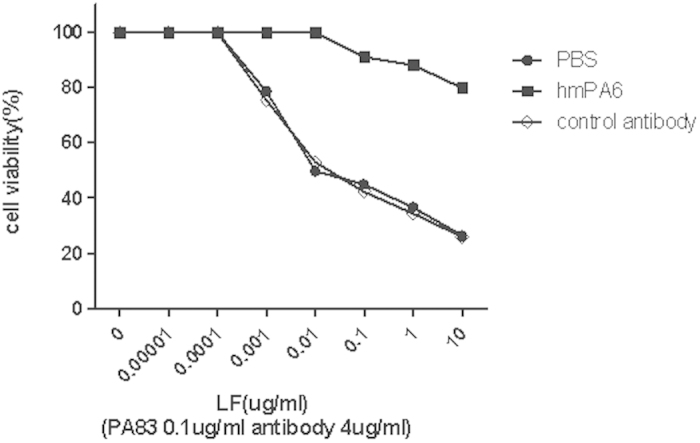
J774A.1 cell survival with hmPA6 treatment. Serially diluted LF was incubated with PA83 and different antibodies for 3 h. Using the AQ assay, cell viability was determined and plotted as survival percentage.

The ability of hmPA6 to protect against LeTx was assessed in J774A.1 cells. hmPA6, PA83, and different concentrations of LF were simultaneously added to cells. Cell viability test results indicated that hmPA6 could completely neutralize LeTx. At 10 μg/mL LF and 0.1 μg/mL PA83, >80% of the hmPA6-treated cells remained viable, while only 26% of the control IgG antibody-treated cells remained viable. At 0.01 μg/mL LF and 0.1 μg/mL PA83, 100% of the hmPA6-treated cells were viable, while only 50% (Fig. 5) of the control cells were.

### Protection of F344 rats

**Figure 6:**
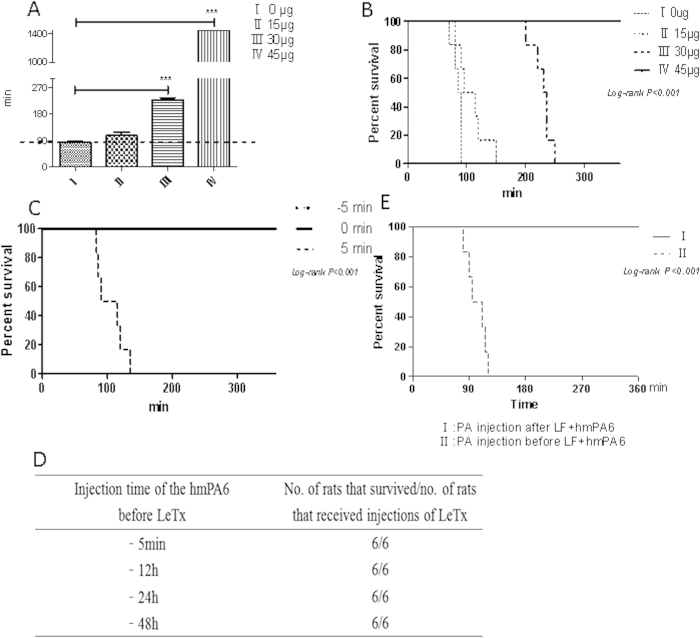
*In vivo* LeTx neutralization assay in F344 rats. **A**. Mean survival time. LeTx and the antibody were simultaneously injected via the tail vein. Group I, 0 μg hmPA6 + 30 μg LeTx; group II, 15 μg hmPA6 + 30 μg LeTx; group III, 30 μg hmPA6 + 30 μg LeTx; group IV, 45 μg hmPA6 + 30 μg LeTx. ***P < 0.001. **B**. Different concentrations of the antibody were injected, and LeTx was injected 5 min later via the tail vein. **C**. For each rat, 45 μg antibody was injected before (−5 min), after (5 min), or simultaneously (0 min) with LeTx. **D**. For each rat, 90 μg antibody was injected at different times before LeTx injection. **E**. Group I, PA (30 μg) was injected 5 min after LF (30 μg) + hmPA6 (45 μg); group II, PA (30 μg) was injected 5 min before LF (30 μg) + hmPA6 (45 μg).

F344 rats were injected hmPA6 antibody via the tail vein either before or after LeTx injection. The survival time of group III was significantly (*P* < 0.001) longer than that in groups I and II. Until the last observation, all group IV rats were alive (Fig. 6A). Rats injected hmPA6 5 min before LeTx showed similar results. A dose of 45 μg antibody protected the rates from death (Fig. 6B), although some symptoms, such as accelerated breathing and lethargy, were observed in one rat. hmPA6 injection before or after LeTx administration protected all rats from developing anthrax (Fig. 6C).

The prophylactic function of hmPA6 was tested by injection of the antibody at different times before LeTx injection. In the groups that received prophylaxis 5 min to 48 h before LeTx injection, 6 rats remained alive (Fig. 6D). When hmPA6 was injected before PA, it protected rats from anthrax death regardless of LF injection time (Fig. 6E).

### Tissue pathology and immunohistochemical analysis

**Figure 7:**
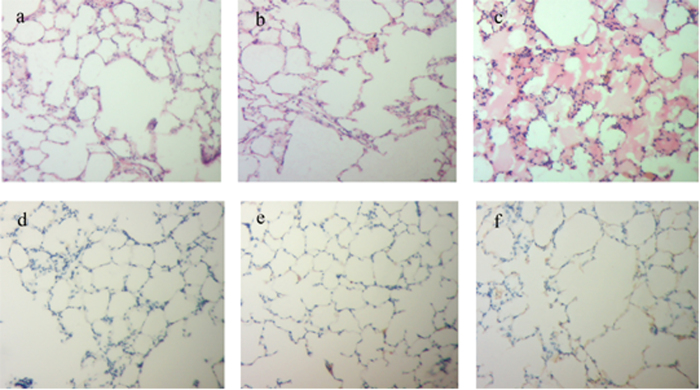
Tissue pathological and immunohistochemical analysis. **a**–**c**. H&E (100×). **d**–**f**. IHC (100×). **a** and **d**. Untreated control rats. **b** and **e**. LeTx + 45 μg hmPA6. **c** and **f**. LeTx alone.

The lung of the rats were pathologically and immunohistochemically examined. H&E staining showed that the local tissue of rats injected only LeTx showed greater alveolar exudation (Fig. 7c) than untreated control rats (Fig. 7a). However, the group that received LeTx + 45 μg hmPA6 (Fig. 7b) showed no significant differences from untreated control rats.

Since anthrax receptors were expressed in some cells including alveolar epithelial cells, cell binding to PA could be positive. When rats were injected with LeTx, the cells could dectect PA. Further, the positive staining was mainly localized to the membrane. A strong positive reaction was found in the group injected only LeTx (Fig. 7f), while a weak positive reaction was found in the group injected LeTx + 45 μg hmPA6 (Fig. 7e). The untreated control group showed a negative reaction (Fig. 7d).

## Discussion

This study revealed two major findings: First, the human/murine chimeric antibody hmPA6 can neutralize LeTx *in vivo* and can be used for prohylaxis before LeTx is released in the blood. Second, since immunization with PA63 produces neutralizing antibody, it can be used to immunize animals. In the present study, we developed four murine mAbs that could well neutralize LeTx *in vitro*, and one clone was selected to form a human/mouse chimeric antibody known as hmPA6. This antibody showed excellent neutralization both *in vitro* and *in vivo*.

The current status of therapeutic mAbs was directed against the major virulence factors: PA, LF, EF and capsule. Although LF could induce cell death, its effective result *in vivo* was unsatisfactory. PA was the critical factor which recognized cell membrane receptors. Then PA was cleaved to active PA63 by Furin which induced LF or EF into cells. Our strategy was to get antibody against active PA63. To immunize the mice, we used PA63, which is formed by the dissociation of PA20 from PA83, instead of PA83, because although PA63 is a part of PA83, these factors may have different structures and expose LF- or EF-binding sites. These differences may in turn lead to the production of completely different antibodies against anthrax *in vivo*. In the present study, we showed that PA63 could induce neutralizing antibodies, as proven through *in vitro* and vivo experiments. On the other hand, it was difficult to obtain active PA63 based on present methods. We screened out positive clones by active PA63 in a different way. Based on traditional ELISA, we coated plates with alive J774A.1 cells overninght at cell culture condition. Then PA83 was added into wells incubated about 2 h. The negative control was cells without PA83 adding. The rest of ELISA procedures was as normal. In this screening system, the active PA63 was more closed to *in vivo* state and could oligomerize to heptamer. In all, more diversity antibodies was generated in the immunization stage and more effective antibodies was obtained in the screening system.

Anthrax, whether resulting from natural or bioterrorist-associated exposure, is a constant threat to human health^23^, and the low incidence of anthrax suggests that large-scale vaccination may not be the most efficient means of controlling this disease. Passive immunization with protective antibodies is therefore considered the primary available biodefense measure^21^, especially in bioterrorist-associated exposure. In the present study, hmPA6 was found to provide good protection in rats challenged with anthrax virulence factors. In the *in vitro* experiment, hmPA6 maintained 100% cell viability with 0.01 μg/mL LF and 0.1 μg/mL PA83, while non-correlated IgG maintained only 50% cell viability. Further, with 10 μg/mL LF and 0.1 μg/mL PA83, hmPA6 maintained >80% cell viability, while the control antibody maintained only 26%. Moreover, in previous study, they often used F344 rats challenged with LeTx (Table 1) before tested with B. anthracis spores. Therefore F344 rats was injected with LeTx vial tail vain. The hmPA6 with a Kd of 0.14 nM protected all rats from death at a concentration of 0.3 mg/kg (45 μg per rat). However, in a previous study, 1.5 mg/kg raxibacumab (of human origin) with a Kd of 2.78 nM was administered 24 h before anthrax lethal toxin administration in F344 rats^24^. We also tested an hmPA6 concentration of 0.6 mg/kg (90 μg per rat) administered 48 h before LeTx injection; this dose also protected all rats. Our findings indicate that hmPA6 may be used as a prophylactic. Other humanized or chimeric mAbs have been examined in other animal model, but the affinity found in the present study is the same as or better than those found previously^32^,^33^. Murine mAbs against PA have been developed, but our chimeric mAb maintains a balance between the high-affinity murine component and the low-immunogenicity human component. Other mAbs of human origin are available but are difficult to produce and are expensive^32^. The chimeric mAb hmPA6 is produced in 293F cells, and the method of production is very convenient.

In the *in vivo* test in the present study, irrespective of whether LF was injected before or after hmPA6, the antibody protected the rats from death provided that it was administered before or simultaneously with PA. This finding indicated that hmPA6 could not prevent LF from binding PA63. Further, no morphological changes were observed in the rats of only injection LeTx, probably because the time between injection and sacrifice was very short (only about 90 mins), and no histological changes occurred in this period. The IHC analysis showed a strong positive result in the group injected only LeTx. However, the group that received LeTx + 45 μg hmPA6 showed a weakly positive reaction. These findings indicate that hmPA6 prevents PA from binding to cell receptors. However, further experiments are required to validate this hypothesis.

Future studies should focus on detailed characterization of this mAb (specificity, toxicity studies, autoantigen testing, etc.). Second, epitope mapping and structure function analysis of hmPA6 should be performed. In the *in vivo* experiment in the present study, we demonstrated that hmPA6 could not interfere with LF binding to PA. Further experimentation is needed to determine the exact mechanism by which hmPA6 neutralizes LeTx. Lastly, more animal tests are required, for example, in which animals are challenged with anthrax spores.

In summary, we reported a human/murine chimeric IgG, namely, hmPA6, which can specifically identify PA with high affinity, neutralize LeTx, and protect macrophages and F344 rats from anthrax-related death. We also showed that PA63 is a good immunogen. From our findings, we believe that once hmPA6 is further characterized, it can be used alone or in combination with other neutralizing mAbs for treatment of anthrax.

## Materials and Methods

### Mouse mAb development

Recombinant PA63 protein (rPA63) was expressed in *Escherichia coli* BL21 by using the pColdII vector and purified by affinity chromatography. Six BALB/c mice aged 7 weeks were intraperitoneally (ip) immunized with rPA63 and adjuvant as described previously^34^. After immunization, the mouse spleen showing the best titer was removed, and splenocytes were extracted and fused with SP2/0 myeloma cells using hybridoma technology^35^. Positive clones were screened by ELISA using active PA63-coated 96-well plates, and subcloning was conducted based on standard protocols. Clonal expansion was conducted with Hybridoma-SFM (Gibco,USA). The cell supernatant was then removed and purified by affinity chromatography with protein G (GE, USA) according to the manufacturer’s purification system.

All experiments involving animals were performed in accordance with the protocols approved by the Animal Care and Use Committee of the National Institute of Allergy and Infectious Diseases, National Institutes of Health, USA.

### Construction of human/mouse chimeric antibody expression vector

Total RNA was extracted from the PA6 hybridoma cells using the TRIzol reagent (Invitrogen), and cDNA was synthesized using reverse transcriptase SuperScript II according to the manufacturer’s instructions. Eukaryotic vectors were constructed by separately cloning PA6 heavy and light variable regions into pTH and pTL, which respectively include constant regions of IgG1 heavy and light chains. Murine variable regions of the heavy (V_H_) and light chains (V_L_) were first amplified by PCR using PA6 cDNA as the template. To obtain V_H_ and V_L_ nucleotide sequences, these chains were cloned into the pMD-18T vector. PCR primers were designed using the In-FusionR HD Cloning Kit (Clontech), and V_H_ and V_L_ were amplified from right-sequenced pMD-18T vectors by using these primers. Finally, V_H_ and V_L_ were separately cloned into linearized pTH and pTL vectors, respectively, by infusion PCR using the In-FusionR HD Cloning Kit. The recombinant pTH/PA6 V_H_ and pTH/PA6 V_L_ vectors were sequenced by Genescript. Sequences were further analyzed using the VBASE2 database (http://www.vbase2.org/).

### Antibody expression and purification

The recombinant vectors were simultaneously transfected into FreeStyle™ 293-F Cells (293F) using 293fectin with the FreeStyle™ 293 Expression System (Invitrogen). Six days after transient transfection, the cell supernatant was harvested and purified by affinity chromatography with protein A (GE, USA) in accordance with the manufacturer’s purification system. The purity of the chimeric antibody (hmPA6) was examined by 10% SDS-PAGE and Coomassie blue staining.

### ELISA

Ninety-six-well enzyme immunoassay plates were coated overnight at 4 °C with 50 μL of rPA63 antigen (2 μg/mL) diluted in 50 mM sodium carbonate buffer (pH 9.6). The plates were blocked and serial two-fold dilutions of hmPA6 were added to the wells (3 wells for each concentration) as the primary antibody. The plates were incubated at 37 °C for 1 h and then washed 3 times with 300 μL of PBS containing 0.05% Tween 20 (PBST). Subsequently, goat anti-human IgG–HRP conjugate (Sigma) was added as the secondary antibody and incubated at 37 °C for 30 min. After color development, the absorbance values of the wells were detected at 450 nm. Non-correlated IgG1 was used as the control. The absorbance values at 450 nm of hmPA6 were plotted using GraphPad Prism software version 5.0 (GraphPad Software, Inc., La Jolla, CA, USA).

### Western blot analysis

The cell lysates of rPA63 recombinant bacteria and *E. coli* BL21 were separately run on a 10% SDS-PAGE gel and then transferred onto a nitrocellulose membrane (Bio-Rad). The membrane was blocked with PBS containing 5% dry milk at 4 °C overnight and then incubated for 1 h at RT with 1:2000 diluted hmPA6 from 1 mg/mL stock. After it was washed 3 times with PBST, the membrane was incubated with a 1:4000 diluted secondary HRP-conjugated goat anti-human antibody (Sigma) for an additional 30 min at RT. Following the same washing procedure, the signal was detected using ECL Western Blot Substrate (Pierce) according to the manufacturer’s instructions.

### Immunoprecipitation

A mixture of PA63 and PA83 was prepared by incubating PA83 [20 mM Tris (pH 8.0) and 150 mM NaCl] with 0.5 μg/mL trypsin (Sigma) for 30 min at 22 °C, followed by addition of 10 μg/mL soybean trypsin inhibitor (Sigma)^14^. Then, the mixture was incubated with 5 μg of hmPA6 at 4 °C and rotated for 3 h. Next, 50 μl protein-A Sepharose (Invitrogen, USA) was added and incubated at 4 °C. The immune complexes that formed were washed 3 times with PBST. Subsequently, 50 μL elution buffer was added to separate these antibody-antigen complexes from protein-A Sepharose. As a negative control, another anti-TLR4 chimeric antibody (generated by our lab) was created using the same protocol. The protein complexes were isolated by running two 10% SDS-PAGE gels; one was transferred onto a nitrocellulose membrane, and the other was stained with Coomassie blue. The nitrocellulose membrane was blocked at 4 °C overnight, incubated with 1:5000 diluted rabbit polyclonal anti-PA antibody (Pierce, USA) for 1 h at RT, washed with PBST 3 times, and reacted with 1:4000 diluted goat anti-rabbit IgG-HRP conjugate (Sigma) for an additional 30 min at RT. The membrane was washed 3 times with PBST, and the hybridization signal was detected using ECL Western Blot substrate. The target bands on SDS-PAGE gel were subjected to mass spectra identification with an ABI 4700 proteomics analyzer and MALDI-TOF/TOF mass spectrometer (Applied Biosystems, Framingham, MA). The mass spectra were then searched within the Swiss-Prot database using the MASCOT search engine (http://www.matrix science.com; Matrix Science, UK).

### Affinity and kinetic assay of antibody

The Biacore X100 System (GE, USA) was used to analyze the affinity and kinetics of the hmPA6 antibody. PA83 was diluted to 25 μg/mL with acetate buffer (10 mM NaAc, pH 4.5) and immobilized on the surface of a CM5 sensor chip (GE, USA) to capture purified mAb, which was diluted in running buffer (10 mM HEPES, 150 mM NaCl, 5 mM EDTA-Na_2_, 0.05% P20; pH 7.4) to achieve different concentrations ranging from 5 to 80 nmol/L. The association time was set up at 180 s and the dissociation time, at 600 s, followed by regeneration with 50 mM glycine–HCl (pH 2.2). Sensograms were evaluated using the Biacore X100 evaluation software.

### *In vitro* LeTx neutralization assay

The *in vitro* LeTx neutralization assay was performed as described previously^29^. Briefly, murine macrophage J774A.1 cells cultured in DMEM containing 10% fetal bovine seru and 1% penicillin/streptomycin were seeded in 96-well plates to 70% confluence. LF was diluted serially in complete medium containing PA and hmPA6. This mixture was applied to the cells (3 wells for each dilution) at the following final concentrations: LF, 0.01 ~ 10,000 ng/mL; PA, 0.1 μg/mL; and hmPA6, 4 μg/mL. The plates were then incubated for 3 h at 37 °C. Untreated cells and cells treated with only LeTx acted as the controls. Cell viability was determined using the AQ assay (Promega, MI) according to the manufacturer’s instructions.

### *In vivo* LeTx neutralization assay

The *in vivo* LeTx neutralization assay was performed using female Fischer 344 (F344) rats weighing between 130 and 160 g. Every rats of each group (n = 6) were injected via the tail vein with a mixture of PA + LF (LeTx) and different amounts of hmPA6 antibody prepared in sterile PBS. Each rat was administered 300 μL of the mixture.

Further, the rats were also treated with different concentrations of the antibody 5 min before exposure to LeTx. For this experiment, they were injected intravenously with PBS or 15, 30, or 45 μg of the antibody before receiving an intravenous injection of LeTx (30 μg PA + 30 μg LF). Additionally, double the complete protection dose of antibody (90 μg) was injected to test its prophylactic ability. The rats were inoculated with 90 μg antibody followed by LeTx administration after different times, from 5 min to 48 h. Two additional experiments were conducted with F344 rats. One group received PA (30 μg) injection 5 min after LF + hmPA6 (30 μg + 45 μg, respectively), while the other received 30 μg PA 5 min before LF + hmPA6 (at the same doses). After injection of LeTx, signs of malaise and death were checked for every 30 min for the first 8 h and then at 16 h and 24 h, followed by twice-daily checks for 1 week.

### Tissue pathology and immunohistochemical examination

The lungs of the F344 rats were embedded in paraffin wax at the Department of Pathology, Nanjing Medical University (Jiangsu, China), using routine methods. Sections (5 μm) were deparaffinized with xylene and then dehydrated in decreasing concentrations of alcohol. Some sections were treated with H&E staining and examined by light microscopy to determine the pathological features of the lung tissues.

For the remaining sections, endogenous peroxidase activity was blocked by incubation with 3% hydrogen peroxidase in Tris-buffered saline. Some of these tissue sections were then incubated with rabbit polyclonal anti-PA primary antibody (Pierce, USA), followed by the EnVision HRP complex (DAKO, Carpinteria, CA). They were then counterstained with hematoxylin QS (Vector Laboratories, Burlingame, CA). The results were analyzed according to the IHC score (IHS) as described previously^36^. Briefly, the IHS was determined by evaluation of both staining density and intensity. Multiplication of the intensity and percentage scores yielded the final IHS. Samples with IHS ≤3 were considered weakly positive, while those with IHS ≥6 were considered strongly positive. The IHC results were evaluated by two independent investigators blinded to the rat groups. In cases of conflict, a pathologist reviewed the cases, and a consensus was reached.

### Statistical analysis of survival data

Kaplan Meier analysis was used for evaluation of survival. Survival data were analyzed using the GraphPad Prism version 4 statistical analysis software (San Diego, CA). A *t*-test was used to compare the mean survival time between groups. A two-tailed log rank test was used to determine the statistical significance of differences between groups. A *P* value of <0.05 was considered statistically significant.

## Additional Information

**How to cite this article**: Xiong, S. *et al.* A Novel Chimeric Anti-PA Neutralizing Antibody for Postexposure Prophylaxis and Treatment of Anthrax. *Sci. Rep.* **5**, 11776; doi: 10.1038/srep11776 (2015).

## Figures and Tables

**Table 1:** Other monoclonal antibodies *in vivo* test.

	mAb	In vivo neutralization	Reference
Anti-LF	LF8	Athymic nude mouse^1^	29
	9A11	Balb/C mouse^1^	28
	10G3, 2E7, 3F6	F344 rat^1^	37
	5B13B1, 3C16C3	F344 rat^1^	30
	IQNLF	A/J mouse^1^	22
	LF10E	F344 rat and A/J mouse^1^	38
	LF11H	F344 rat^1^	38
Anti-PA	Abthrax	F344 rat^1^, rabbit^2^ and monkey^2^	24
	AVP-21D9	F344 rat^1^ and rabbit^2^	25
	IQNPA	A/J mouse^2^	22
	MDX 1303	Rabbit^2^ and monkey^2^	26

^1^Animals challenged with LT.

^2^Animals challenged with B. anthracis Ames spores.
